# Trends in patients’ overall satisfaction with healthcare delivery in Accra, Ghana

**DOI:** 10.4102/phcfm.v11i1.1884

**Published:** 2019-09-17

**Authors:** Stephen T. Odonkor, Charles Frimpong, Emmanuel Duncan, Carolyn Odonkor

**Affiliations:** 1 School of Public Services and Governance, Ghana Institute of Management and Public Administration, Accra, Ghana; 2 Ghana Space Science and Technology Institute, Ghana Atomic Energy Commission, Accra, Ghana; 3 Finance Department, Methodist University College, Accra, Ghana; 4 Young African Leadership Initiative, Regional Leadership Centre, Ghana Institute of Management and Public Administration, Accra, Ghana

**Keywords:** Satisfaction, Healthcare, Patients, Quality, Expectation and rights

## Abstract

**Background:**

Patient satisfaction represents a key marker for the quality of healthcare delivery and is critical for smooth functioning of the healthcare system.

**Aim:**

The aim of this study was to determine the levels of patient satisfaction with the quality of care they receive, and thus identify the key factors that influence patients’ overall satisfaction with healthcare.

**Setting:**

The study was conducted across seven healthcare facilities in Greater Accra region.

**Methods:**

The study employed a cross-sectional design to obtain data from 417 respondents between 01 November 2017 and 31 January 2018. Patient satisfaction within the context and setting of this study refers to the extent to which patients are happy with the healthcare services they receive. Statistical analysis was performed using Statistical Package for the social Sciences (SPSS) version 23.

**Results:**

Female patients constituted 66.7% of the respondents, while 33.3% were male patients. Most of the participants had health insurance coverage (95.2%). Overall, 69.5% of the patients were satisfied with the level of care, 29.3% were somewhat satisfied and 1.2% were not satisfied. Female patients (86.0%) were more satisfied with the level of care they received compared to male patients (61.9%). Majority of the female patients (87.8%) indicated that they were treated with courtesy and respect. However, more than a half (51.8%) of male patients indicated they were not treated with courtesy and respect. Patient satisfaction negatively correlated with social status and age of the patients.

**Conclusion:**

Measurement of patients’ satisfaction is important for healthcare delivery. It was clear from this study that there is still a gap in improving and managing patients’ satisfaction and expectation. All stakeholders must get involved to ensure timely and satisfactory healthcare delivery to all patients.

## Introduction

The importance of patient satisfaction in healthcare delivery cannot be overemphasised. Patient satisfaction is a set of attitudes and perceptions of patients towards health services.^1,2^ It is the degree to which an individual regards healthcare as useful, effective and beneficial.^3,4^ In other words, it is the judgement of patients about their needs and expectations met by the care provided,^5,6,7^ or an evaluation based on the fulfilment of expectations of the user.^4^ Thus, satisfaction is a psychological state that results when the emotion surrounding disconfirmed expectations is coupled with consumer’s prior feelings about the consumption experience.^8^ It is actually determined by the interplay of two factors: patient expectations and experience of the real services. If the performance falls short of expectations, there is dissatisfaction, and if it matches the expectations, then vice versa.^9^ Patient satisfaction is therefore a match of expectations with experiences of the patient during a treatment process.^10^

Patient satisfaction represents a key marker for the quality of healthcare delivery and this internationally accepted factor needs to be studied repeatedly for smooth functioning of the healthcare system.^11,12^ It is worth noting that the World Health Organization in 1989 directed all member states to introduce regular assessment of the quality of their health services and to establish principles for quality assurance programmes.^13^ It is therefore important to evaluate the level, scope and quality of the delivery scheme for possible policy recommendation. A better appreciation of the factors pertaining to client satisfaction would result in implementation of custom made programmes according to the requirements of the patients, as perceived by patients and service providers.^14^ Patients are the best judges and their inputs are critical in the overall improvement of quality healthcare delivery system.^15^

In Ghana, the Institutional Care Division of Ghana Health Service has direct responsibility of ensuring healthcare quality. A qualitative analysis of satisfaction with medical services was conducted in 1997 and 2003 as part of the Core Welfare Indicator Questionnaire. It was found that satisfaction had increased from 57.0% in 1997 to 78.6% in 2003, indicating a 21 percentage point increase. However, the level of satisfaction was not scaled but simply defined for persons who consulted health practitioners and cited no problem with the health system.^16,17^

Several gaps exist within the patient satisfaction domain in Ghana. Although some work has been conducted, work still remains. For example, a holistic assessment of satisfaction across districts regarding the services of a given health provider is virtually non-existent. Yet, it is critical to have integrated information about patient satisfaction in order to aid policy directions and the likes. Secondly, previous studies have investigated into detail patient’s demographic characteristics in relation to patient satisfaction. However, the characteristics such as age, educational level, health status and amount of information conveyed by the health provider are significant predictors of healthcare satisfaction.^18^

The aim of this article is to first determine the levels of patient satisfaction with the quality of care they receive, and thus identify the key factors that influence patients’ overall satisfaction with healthcare. To achieve our objective, we sought to answer four main questions: (1) What are the satisfaction levels of patients receiving healthcare service? (2) Are there differences in service experiences of healthcare satisfaction between insured and non-insured patients? (3) What are the key factors that influence patients’ overall satisfaction with healthcare? and (4) Does socio-demographic characteristics influence a patient’s level of healthcare satisfaction?

## Methods

### Description of the study location

The study was conducted in the Greater Accra region, which lies on the south-eastern part of the country. The region occupies a total land area of 3245 km^2^, which makes it the smallest region of the 10 political regions in Ghana in terms of land size. It has a population density of 1235.8 people per square kilometre. The region is 90.5% urban, with an annual urban growth rate of 3.1%. It experiences more inflows of people from other parts of the country than people moving out the region. The doctor to patient ratio in the region is 1:10 450. Access to healthcare in the region is limited despite progress made by government in improving healthcare; public hospitals remain overcrowded and underfunded.

### Study design and sample size

The study employed a cross-sectional design to obtain quantitative data. The study was carried out in seven healthcare facilities in the Greater Accra region of Ghana. A total of 550 questionnaires were distributed across the seven healthcare facilities in the Greater Accra region based on the proportion of the human population within the districts in which the health facilities are located. However, only 417 questionnaires were completely filled and returned, which gave a response rate of 75.81%.

### Sampling technique

The study utilised a stratified sampling technique. The total number of respondents in the seven healthcare facilities was obtained through a proportional sampling to size method, based on population census figures within those districts. Thus, in selecting the respondents, sampling proportionate to size was used to determine the number of respondents to be interviewed from each healthcare facility. At the healthcare facility, patients who were present at the facility were considered for the study.

### Data collection and procedure

Data collection study took place between November 2017 and January 2018. A standardised structured questionnaire designed to meet the objectives of this research was used for data collection. Field inspection of questionnaire data was carried out daily after the interview was conducted, and any errors were immediately verified and corrected. The survey instrument consisted of 23 questions, including socio-demographic characteristics of respondents, respondents’ experiences at the health facility, respondents’ overall satisfaction of the healthcare received and respondents’ ratings of their experience at the health facilities. The final instrument was administered to the subjects via a self-administered questionnaire method. It took approximately 25–35 minutes to complete the instrument.

Five experts in health measurement and evaluation assisted with the determination of face validity of the instrument. The average overall face validity was equal to 95%. Test reliability for internal consistency was performed using the Cronbach’s alpha test. It was equal to a reliability coefficient of 0.87, which is adjudged high reliability. Cronbach’s alpha is a measure that assesses the internal consistency of a set of scale or test items to ensure that they are all consistent in measuring the same attributes under investigation.

### Data handling and analysis

The data were entered into a spreadsheet and later exported to SPSS version 23 and coded for analysis. The analysis included both descriptive and inferential statistics. Descriptive statistics (frequencies, means and standard deviations) were used to describe the variables of interest. Univariate analysis was used in obtaining the frequency of socio-demographic characteristics and other discrete variables of the study population. Data were analysed by contingency table except for *t*-tests as appropriate for continuous data (e.g. age). The chi-squared (*χ*^2^) tests were used to assess the bivariate relationships between these factors as well as for differences in proportions and for other categorical variables. Cramer’s *V* = 0.208 exact test was used to determine the strength of relationships. Post-hoc analysis was also carried out.^19,20^ All statistical tests were two-tailed and an alpha value of 0.05 or less was considered statistically significant.

### Ethical considerations

Prior to data collection, respondents’ verbal consent was sought. The respondents were informed about the purpose of the study and were made aware that participation was voluntary and refusal to participate in the study would not affect them in anyway. They were assured of confidentiality and informed that they could withdraw from the study at any time and were at liberty not to answer any question they did not want to. All the respondents were advised that completing the survey implied informed consent to use the data for research purposes. In addition, all personal identifiers were removed in the summary data to ensure confidentiality.

## Results

In total, 417 respondents were included in the analyses. Table 1 presents the demographic characteristics of the respondents. A higher proportion of respondents were women (*n* = 278), representing 66.7%, while 33.3% were men. The age of respondents ranged from below 29 to above 61 years, with the majority being below 29 years (69.5%).

**TABLE 1:** Demographic characteristics of respondents.

Variable (*n* = 417)	Male		Female		Significance level		
	*N*	%	*N*	%	*X*^2^	*p*	df
**Age (years)**							
≤ 29	98	33.8	192	66.2	-	-	-
30–40	19	32.8	39	67.2	-	-	-
41–50	8	19.0	34	81.0	-	-	-
51–60	12	52.2	11	47.8	-	-	-
≥ 61	2	50.0	2	50.0	-	-	-
Total	139	33.3	278	66.7	8.067	0.089	4
**Religion**							
Christian	126	32.6	260	67.4	-	-	-
Islam	5	29.4	12	70.6	-	-	-
Traditionalist	8	57.1	6	42.9	-	-	-
Total	139	33.3	278	66.7	3.772	0.152	2
**Ethnicity**							
Akan	104	30.2	240	69.8	-	-	-
Ga-Adangbe	13	40.6	19	59.4	-	-	-
Mole-Dagbon	3	60.0	2	40.0	-	-	-
Ewe	10	43.5	13	56.5	-	-	-
Others	9	69.2	4	30.8	-	-	-
Total	139	33.3	278	66.7	12.458	0.014	df = 4
**Marital status**							
Single	106	31.3	233	68.7	-	-	-
Married	27	48.2	29	51.8	-	-	-
Divorced	5	33.3	10	66.7	-	-	-
Widow or widower	1	14.3	6	85.7	-	-	-
Total	139	33.3	278	66.7	7.374	0.061	3
**Education**							
Basic	8	40.0	12	60.0	-	-	-
Secondary	13	33.3	26	66.7	-	-	-
Tertiary	116	32.6	240	67.4	-	-	-
Others	2	100.0	0	0.0	-	-	-
Total	139	33.3	278	66.7	4.490	0.213	3
**Employment status**							
Self-employed	19	35.2	35	64.8	-	-	-
Employed	31	14.4	185	85.6	-	-	-
Unemployed	89	60.5	58	39.5	-	-	-
Total	139	33.3	278	66.7	84.084	0.000	2
**First visit to health facility**							
Yes	71	55.5	57	44.5	-	-	-
No	68	23.5	221	76.5	-	-	-
Total	139	33.3	278	66.7	40.723	0.000	1
**Health insurance**							
Yes	130	32.7	267	67.3	-	-	-
No	9	45.0	11	55.0	-	-	-
Total	139	33.3	278	66.7	1.287	0.257	1
**Sector of Employment**							
Formal sector	56	26.7	154	73.3	-	-	-
Informal sector	29	19.6	119	80.4	-	-	-
Not applicable	54	91.5	5	8.5	-	-	-
Total	139	33.3	278	66.7	−106.678	0.000	2
**Occupation**							
Teacher or lecturer	12	15.4	66	84.6	-	-	-
Banker	10	20.8	38	79.2	-	-	-
Driver	0	0.0	12	100.0	-	-	-
Others†	6	31.6	13	68.4	-	-	-
Student	96	51.6	90	48.4	-	-	-
Trader	15	20.3	59	79.7	-	-	-
Total	139	33.3	278	66.7	54.359	0.000	5
**Social status**							
Upper class	19	13.7	30	10.8	-	-	-
Middle class	114	82.0	240	86.3	-	-	-
Lower class	6	4.30	8	2.9	-	-	-
Total	139	33.3	278	66.7	1.428	0.490	2

† , This included informal workers such as farmers, construction workers and so on.

The majority of respondents were Christians (92.6%), followed by Muslims (4.1%) and Traditionalists (3.4%). In terms of ethnicity, respondents identified themselves as Akans (82.5%), Ga-Adangbes (7.7%), Mole-Dagbons (1.2%), Ewes (5.5%) and from other ethnic groups not stated (3.1%).

Overall, 81.3% of all respondents reported that they were single at the time of the survey, 13.4% reported that they were married and 3.6% reported that they were divorced. A small percentage reported they are widowed (1.7%). The respondents were relatively well educated, with 85.4% having a tertiary education.

In terms of employment, half of the respondents were employed (51.8%) and almost equal number of respondents (50.4%) were working in the formal sector. Almost half (48.2%) were either self-employed or unemployed, and 35.5% of the respondents were working in the informal sector. The majority of respondents (69.3%) had visited the health facility at least once, while 30.7% were visiting for the first time.

Most of the participants had health insurance coverage (95.2%) and reported that they belonged to the middle class in terms of social status (84.9%).

The overall level of satisfaction of care received by patients as obtained from the data analysis is detailed below in Tables 2 and 3. Overall, 69.5% of the patients were satisfied with the level of care, 29.3% were somewhat satisfied and 1.2% were not satisfied. Majority of female patients (86.0%) were satisfied with the level of care they received as compared to the majority of male patients (61.9%) being somewhat satisfied with the level of care (Table 2).

**TABLE 2:** Respondents’ overall satisfaction of the care they received.

Variable	Satisfied		Somewhat satisfied		Not satisfied		Significance level		
	*N*	%	*N*	%	*N*	%	*X*	*p*	Cramer’s V
**Gender**							108.264	0.000	0.510
Male	51	36.7	86	61.9	2	1.4	-	-	-
Female	239	86.0	36	12.9	3	1.1	-	-	-

**TABLE 3:** Relationship between satisfaction and health insurance.

Insurance status	Satisfied		Somewhat satisfied		Not satisfied	
	*N*	%	*N*	%	*N*	%
Insured	282	71.0	110	27.7	5	1.3
Non-insured	8	40.0	12	60.0	0	0.0

Significance level: *X* = 9.675; *p* = 0.008; Cramer’s V = 0.152.

For patients who were insured, majority were satisfied with the level of care (71.0%), 27.7% were somewhat satisfied and 1.3% were not satisfied. For patients who were uninsured, the majority were somewhat satisfied (60.0%), 40.0% were satisfied and none indicated to be unsatisfied with the level of care received (Table 3).

Table 4 shows patients’ experience at various health facilities under study. From the table, it can be seen that the majority of patients recognised exceptional service provided by hospital staff (81.1%) and indicated that hospital staff listened carefully to them when they had a question or concern (86.1%). The same trend is seen in positive response to questions on examinations being performed at the right time (72.9%), privacy during examinations (85.1%), non-inducement of workers before being examined (93.5%) and satisfaction with reception (96.2%). However, the majority of patients (61.4%) indicated that they were not given adequate instruction or explanation before examinations were carried out on them.

**TABLE 4:** Respondents’ experiences at the health facility.

Descriptions	Male				Female			
	Yes		No		Yes		No	
	*N*	%	*N*	%	*N*	%	*N*	%
Is there anyone you would like to recognise for exceptional service?	106	25.4	33	7.9	232	55.6	46	11.0
Did the staff listen carefully to you if you had a question or concern?	113	27.1	26	6.2	246	59.0	32	7.7
Did the staff treat you with courtesy and respect?	67	16.1	72	17.3	244	58.5	34	8.2
Was your examination performed at the right time?	73	17.5	66	15.8	231	55.4	47	11.3
Were you given privacy during your examination?	114	27.3	25	6.0	241	57.8	37	8.9
Were you given adequate instruction or explanation before the examination was carried out?	62	14.9	77	18.5	99	23.7	179	42.9
Was the waiting area comfortable?	61	14.6	78	18.7	153	36.7	125	30.0
Were you directed to where to be at any point in time?	107	25.7	32	7.7	103	24.7	175	42.0
Did you induce the workers before you were examined?	12	2.9	127	30.5	15	3.6	263	63.1
Were you satisfied with the way you were received?	127	30.5	12	2.9	274	65.7	4	1.0

The majority of female patients (87.8%) indicated that they were treated with courtesy and respect. On the contrary, a higher proportion of male patients (51.8%) indicated otherwise. We also see differences in responses on receiving appropriate directions and comfort of waiting area among the two groups (Table 4).

Table 5 shows the correlation between patient satisfaction and selected demographic variables. From the table, it can be observed that patient satisfaction correlated positively with ethnicity and marital status. However, patient satisfaction negatively correlated with social status and age of the patients.

**TABLE 5:** Correlation matrix and descriptive statistics of patient satisfaction and demographics.

Variables	PS	AG	GE	RE	ET	MS	ED	ES	HS	SS
Patient satisfaction (PS)	1.000	−0.099*	0.477**	0.118*	0.007	0.009	0.071	0.302**	0.130**	−0.012
Age (AG)	−0.099*	1.000	0.009	0.057	0.0450	0.478**	−0.285**	−0.311**	−0.026	0.168**
Gender (GE)	0.477**	0.009	1.000	0.076	0.159**	0.037	−0.013	0.302**	0.056	−0.018
Religion (RE)	118.000*	0.057	0.076	1.000	−0.028	0.044	0.039	0.027	−0.060	−0.003
Ethnicity (ET)	0.007	0.045	0.159**	−0.028	1.000	0.154**	−0.091	−0.012	0.071	−0.188**
Marital status (MS)	0.009	0.478**	0.037	0.044	0.154**	1.000	−0.481**	−0.229**	−0.040	−0.063
Education (ED)	0.071	−0.285**	−0.013	0.039	−0.091	−0.481**	1.000	0.232**	−0.029	−0.081
Employment status (ES)	0.302**	−0.311**	0.302**	0.027	−0.012	−0.229**	0.232**	1.000	0.043	−0.117*
Health insurance (HS)	0.130**	−0.026	0.056	−0.060	0.071	−0.040	−0.029	0.043	1.000	−0.069
Social status (SS)	−0.012	0.168**	−0.018	−0.003	−0.188**	−0.063	−0.081	−0.117*	−0.069	1.000
Mean	1.320	27.700	1.330	1.110	1.390	1.260	2.820	2.220	1.050	1.920
Standard deviation	0.491	11.342	0.472	0.405	0.985	0.604	0.507	0.658	0.214	0.380
Kurtosis	−0.093	3.438	−1.504	14.269	5.587	7.153	5.817	−0.750	16.107	3.278

* , Correlation is significant at the 0.05 level (2-tailed);

** , Correlation is significant at the 0.01 level (2-tailed).

## Discussions

The aim of this study was to determine the levels of patient satisfaction with the quality of care they receive, and thus identify the key factors that influence patients’ overall satisfaction with healthcare. The importance of patient satisfaction within the healthcare delivery system cannot be overemphasised. It is considered as an important outcome of the quality of healthcare.^21^ Getting views of the patients on the care services is a much realistic tool to evaluate and improve the healthcare services because it is based on direct experiences of the users.^22,23,24^

In this study, we found that patients were satisfied (69.5%) with the quality of care provided from the seven hospitals. This is similar to findings of patients’ satisfaction studies conducted in a regional hospital in Sunyani, Ghana and a clinic in Glasgow, United Kingdom, where the overall satisfaction of patients with quality of service provided was good.^25,26^ However, this is contrary to studies conducted in Tanzania, whereby a higher proportion of patients were dissatisfied with the quality of care provided.^27,28^

This study revealed that female patients’ satisfaction with level of quality of care is higher than that of male patients. Male patients perceived the quality of care received to be somewhat satisfactory (Table 2). A major reason for this observation may be attributed to the fact that men tend to be more impatient and concerned with the speed of the healthcare process than women. These observations suggest the need to target healthcare satisfaction and quality efforts differently for women and men. Thus, the speed of the healthcare process as well as interpersonal aspects of the care process with care providers may be most critical in improving male satisfaction.

The patients’ level of satisfaction was also found to significantly differ by health insurance status. A higher proportion of insured patients were satisfied with the level of healthcare received compared to the uninsured (71% vs. 40%). This was similar to findings from a patient satisfaction study conducted in Sunyani^29^ and could be attributed to quality of consultation given to each group of patients, waiting times and friendliness of staff towards each group of patients.^29^

**FIGURE 1:**
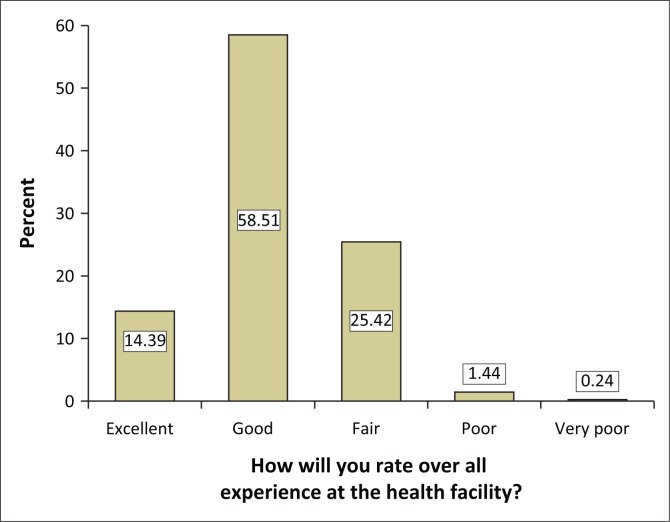
Respondents’ ratings of their experience at the health facilities.

Overall, the majority of patients were generally satisfied with their experience at the health facility (Figure 1). The results from the study demonstrate that the factors that influence overall satisfaction with quality of care are attentiveness to patient’s concern, timeliness of care, professionalism of healthcare staff, respect for patient privacy, comfort of patients and adequate patient knowledge of care being administered. However, there were negative responses from patients about their experiences in all seven hospitals, which revealed that dissatisfaction existed in all factors that influence patient experience and overall satisfaction with quality of care. The negative responses indicated that all patients’ expectations generally were not met. These findings suggest that there is more room for all seven hospitals to improve service quality in relation to these factors.

Generally, it is worth noting that the participants were highly educated, mostly working and had health insurance. These factors could increase the patient expectation for healthcare services and thus their satisfaction with the same as well.

Understanding and measurement of service quality and patients’ satisfaction from the patient’s point of view is important for healthcare delivery because it forms an integral part of the provision of a better and more focused quality service for patients.^25^ Our findings can contribute to the understanding of how patients perceive certain aspects of quality of the healthcare services and feedback from surveys of this nature can be of considerable policy significance.

## Limitations

This study utilised a cross-sectional design, which may present difficulties in ascertaining the direction of causality between the variables analysed. Therefore, caution needs to be taken in the interpretation of the findings with regard to causality. The study might be vulnerable to reporting bias, response bias and selection bias. However, we do not think that this would be a big problem in our study because we used a standardised questionnaire.

## Recommendations

A follow-up study is recommended to be conducted among healthcare providers to assess the challenges they face with meeting expectations of patients and improving patient experience. This is important because if patient’s level of satisfaction on quality of care does not meet their standards, patients may decide to seek treatment somewhere else out of the formal health system that may lead to poor health seeking behaviours resulting in poor initial uptake of services, poor adherence, poor retention of services and at the end, this may contribute to high morbidity and mortality.

Finally, in future studies, the factors for patient satisfaction should be suggested by the patients themselves and not just part of a questionnaire, where the factors have already been mentioned. It is recommended that future studies start with qualitative data to identify patient satisfaction factors, and then a follow-up study to develop a validated optimal tool for patient satisfaction.

## Conclusion

This study sought to determine the trends in patient satisfaction with the healthcare they receive, and thus identify the key factors that influence patients’ overall satisfaction with healthcare. It was clear from this study that there is still a gap in improving and managing patients’ satisfaction and expectation. All stakeholders must get involved to ensure timely and satisfactory healthcare delivery to all patients. Training on customer service for healthcare workers is important.
